# Supplementary figures and images for: Comparative Analysis of Gut Microbiota Diversity Across Different Digestive Tract Sites in Ningxiang Pigs
Source: Animals (Basel). 2025 Mar 25;15(7):936. doi: 10.3390/ani15070936 (PMC11987976; doi:10.3390/ani15070936)

A

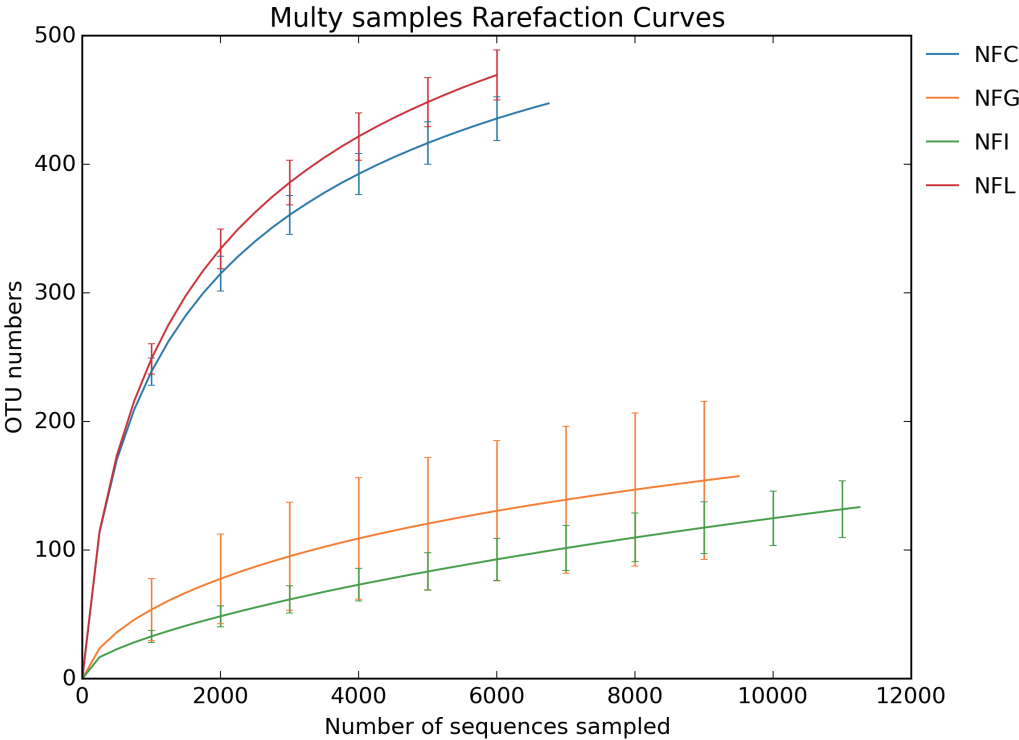

B

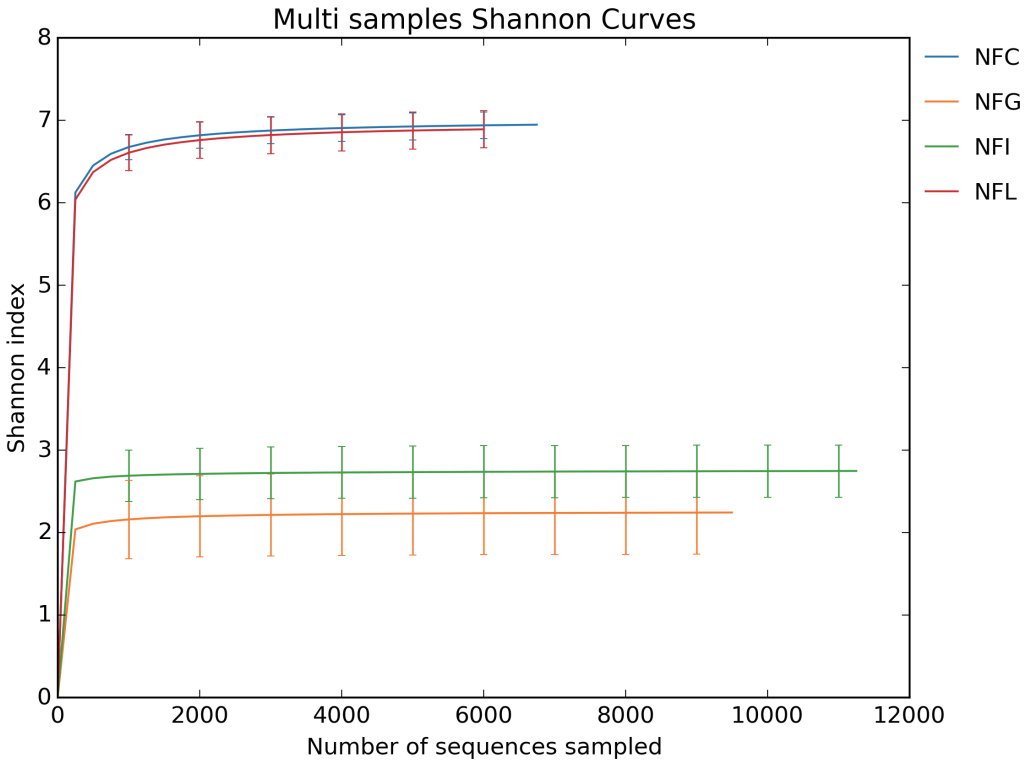

C

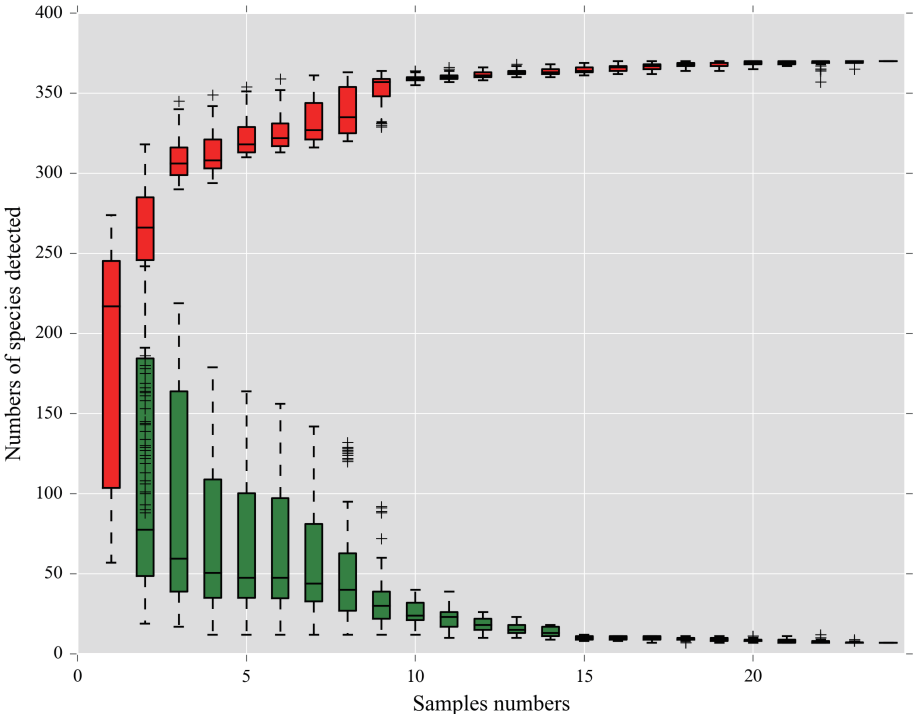

Supplement: Supplementary file 1 [file animals-15-00936-s001.zip › Supplementary FigureS1.pdf]
